# From Adult Morphology to Developmental Hypothesis: Variation of the Adult Lateral Wrist Extensors—A Developmental Viewpoint

**DOI:** 10.3390/jfmk11020172

**Published:** 2026-04-24

**Authors:** Dimo S. Stoyanov, Tsvetomir E. Kachovski, Kamelia Bratoeva, Anton B. Tonchev, Emil G. Kovachev, Stoyan P. Pavlov

**Affiliations:** 1 Department of Anatomy and Cell Biology, Faculty of Medicine, Medical University-Varna, 9000 Varna, Bulgaria; anton.tonchev@mu-varna.bg (A.B.T.); stoyan.pavlov@mu-varna.bg (S.P.P.); 2 Department of Obstetrics and Gynecology, Faculty of Medicine, Medical University-Varna, 9000 Varna, Bulgaria; tsvetomir_kachovski@abv.bg (T.E.K.);; 3 Department of Physiology and Pathophysiology, Medical University-Varna, 9000 Varna, Bulgaria; kamelia.bratoeva@mu-varna.bg; 4 Vascular Biology Research Group (RenEVA), Research Institute, Medical University-Varna, 9000 Varna, Bulgaria

**Keywords:** upper limb development, radial wrist extensor variability, interconnecting tendons, anatomy, variations

## Abstract

**Background:** Anatomical variations are inevitable part of studying the human body. Very often, muscles of the limbs may show atypical attachments, extra or fewer muscle bellies. These variations are likely rooted in limb development. Our goal was to thoroughly study and describe the variations in the lateral wrist extensors. Our initial goal was to attempt to explain the developmental processes that occur before the formation of these variations, with a focus on the interconnecting tendons. **Methods:** We used a standard dissection technique, paying extra attention to the space between the two radial wrist extensors to properly visualize interconnecting tendons. Taking advantage of the chi square test, we compared the observed vs the expected random distribution of interconnecting tendons. **Results:** In this article, we systematically studied the variations in the interconnecting tendons of the lateral carpal extensors in 58 upper limbs of our cadaver donors used for the education of medical students. The main variation we found is interconnecting tendons between the extensor carpi radialis longus and extensor carpi radialis brevis. The insertion and origin of the interconnecting tendons were consistent: it either originates from the middle of the ECRB body and inserts medial to ECRL tendon or it originates from the ventral side ECRL and inserts ventral to the ECRB tendon. We supplemented them with two dissections of fetal upper limbs (at GW 12 and GW 17). Statistical analysis of the distribution of single vs double interconnecting tendons suggests that they are dependent events, consistent with literature data. **Conclusions:** Based on our observations and the literature, we propose that oblique muscle division and a mismatch between the muscle fission plane and the initial distal tendon fission plane may result in the observed phenotype. We also suggest that the origin of the extra numerary tenons form ECRL body may play a role when choosing which one to mobilize for tendon transfer.

## 1. Introduction

The radial wrist extensors consist of two muscles: the m. extensor carpi radialis longus (ECRL) and the m. extensor carpi radialis brevis (ECRB). These muscles attach to the bases of the second and third metacarpal bones (OM II and OM III) [1]. Some of the most common variations in this muscle group include accessory muscles, accessory tendons, variations in the muscle origin, and, lastly, muscle agenesis. The major variation type we focus on is interconnecting tendons (ITs). They can arise from the ECRL, the ECRB, or both, and they cross one another beneath the dorsal antebrachial muscles of the thumb (namely, m. extensor pollicis longus, m. extensor pollicis brevis, and m. abductor pollicis longus).

From a developmental standpoint, muscles and their corresponding tendons have separate origins. Tendons of the extremities are derived from the lateral plate mesoderm while muscle cells originate from the hypaxial part of the dermatomyotomes [2,3,4]. It is worth mentioning that there are some tendon progenitor cells in the somites, but they are responsible for the intrinsic back muscles and intercostal muscle tendons [5].

The muscle progenitors in the hypaxial dermatomyotome express the transcription factor Pxa3, and limb muscles do not form in its absence [6,7]. On the other hand, scleraxis (Scx) is the key marker for tendon precursor cells, and severe defects in force-transmitting tendons are observed in knockout animals [8,9]. In the adult limbs, separate muscles are formed by cleavage from a pre-existing common muscle primordium. The cleavage site starts in its proximal regions and proceeds distally, although, in rare cases, the reverse is possible [3,10].

On the other hand, tendon fission proceeds differently. The distal portion of the tendon, where no muscles are located, has autonomous development, and separate tendons form, while its proximal part appears as one solid tendinous plate. The subsequent development and segregation of this common plate depend on establishing muscle contact and are guided by it; therefore, tendon fission proceeds in the opposite direction, distally to proximally [3,11]. If no muscle contact is established, the tendons will degenerate starting from the proximal end [12,13].

Our main objective was twofold: propose a hypothesis for the development of the lateral wrist extensor by leveraging anatomical variations and systematize the morphological characteristics of the proximal and distal attachments of the interconnecting tendons [14]. We analyzed the variations we observed across fifty-eight dissected upper limbs. Their insertions were always ventral to the main tendon of the ECRB or medial to the ECRL tendon, consistent with the report of Albright et al. (1978) [14]. We divided our observations in tree groups allowing us to assess their distribution. The groups are as follows: 1. An interconnecting tendon emerging from ECRL and inserting into ECRB (L-to-B), 2. An interconnecting tendon emerging from ECRB and inserting into ECRL (B-to-L), and 3. Both interconnecting tendons present on the same upper limb (Both). We additionally describe a consistent anatomical pattern of the interconnecting tendons’ origin: they typically arose either from the ventral portion of the ECRL or the mid-lateral surface of the ECRB. To our knowledge, this specific anatomical consistency has not been previously documented. One exceptional case featured an interconnecting tendon from the ECRB that split equally, originating from its dorsal portion—a morphological pattern distinct from all other specimens in our study.

## 2. Materials and Methods

### 2.1. Cadaver Preparation

Cadaveric donors were fixed in an aqueous solution of formalin (1.2%), ethanol (66%), glycerine (9%), and salicylic acid (0.5%). Limbs were routinely dissected during the Human Anatomy course for medical students at the Medical University of Varna. Human foetuses were fixed in 4% PFA and submerged in a 30% sucrose, 0.1% sodium azide solution for long-term storage.

### 2.2. Dissection

All dissections were performed and imaged under a stereomicroscope. Adult limbs were dissected after fixation and foetal limbs after sucrose treatment. Limbs that had traces of severe damage in ERCL and ECRB from their usage as teaching material were discarded. A total of 28 left and 30 right limbs were dissected.

### 2.3. Statistical Analysis

After obtaining the observed distribution of interconnecting tendons, we calculated the probabilities of both interconnecting tendons being present in the same upper limb if they were independent events by multiplying the probabilities of the single events (see results Section 3.5). The goodness-of-fit test between the observed and expected distributions was performed using a multinomial Pearson’s χ^2^-test. Due to the small number of expected frequencies for some of the groups, the statistical significance of the test was evaluated via a Monte Carlo simulation of *p*-values based on 2000 replications. The statistical analysis was performed in R Language v.4.5.3 and Environment for Statistical Computing and Graphing [15].

## 3. Results

**Figure 1:**
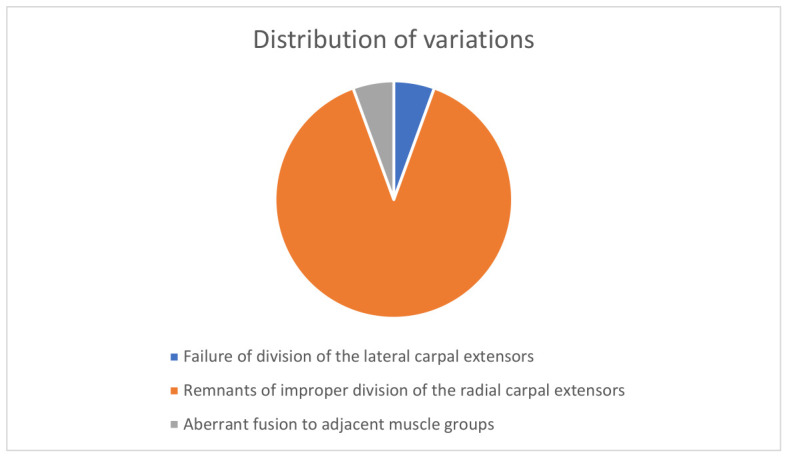
Distribution of variation groups amongst all variable cases.

The dissections were performed as part of educational anatomical courses for medical students. Our initial hypothesis was that we could use the observable variations in adult human specimens to indirectly reconstruct the developmental process. To this end, we examined the morphology of the extensor carpi radialis muscles in 58 limbs, identifying anatomical variations in 17 specimens (32.8%) and 18 variations in total (one specimen had two coinciding anatomical variations). We categorized these 17 cases into three distinct groups based on a review of the existing literature and our own morphological observations: 1. Failure of division of the lateral carpal extensors; 2. Remnants of improper division of the radial carpal extensors (including supernumerary tendons and accessory muscles); 3. Aberrant fusion to adjacent muscle groups. Among these variable cases, the most prevalent variation, occurring in 90% (16/18 variations), involved supernumerary tendons, while the remaining cases were equally divided between failed separation of the muscle bodies (5%; 1/18) and an aberrant fusion between the muscles of the lateral and posterior compartments (5%; 1/18), summarized in Figure 1.

### 3.1. Failure of Division of the Lateral Carpal Extensors

**Figure 2:**
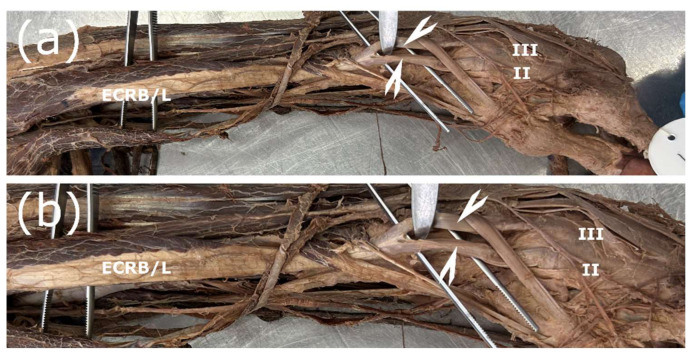
Fusion of ECRL and ECRB (left arm). (**a**,**b**) The ECRL and ECRB have a common muscle body. Division only starts at the level of the tendon (shown with arrowheads). ECRB/L—m. extensor carpi radialis brevis/longus, II—second metacarpal bone, III—third metacarpal bone.

In total, this pattern was present in one case, comprising 1.7% of all studied upper limbs, or 5% of all observed variations. The muscle bodies of the ECRL and the ECRB were fused. One common tendon connected to the muscle and later bifurcated distal to the m. abductor pollicis longus. The two tendons were attached at the expected sites at the base of OM II and OM III. The proximal origin of the common muscle body was from both the lateral epicondyle and the lateral epicondyle ridge—the classical anatomical origins of the ECRL and ECRB (Figure 2).

### 3.2. Remnants of Improper Division of the Radial Carpal Extensors

**Figure 3:**
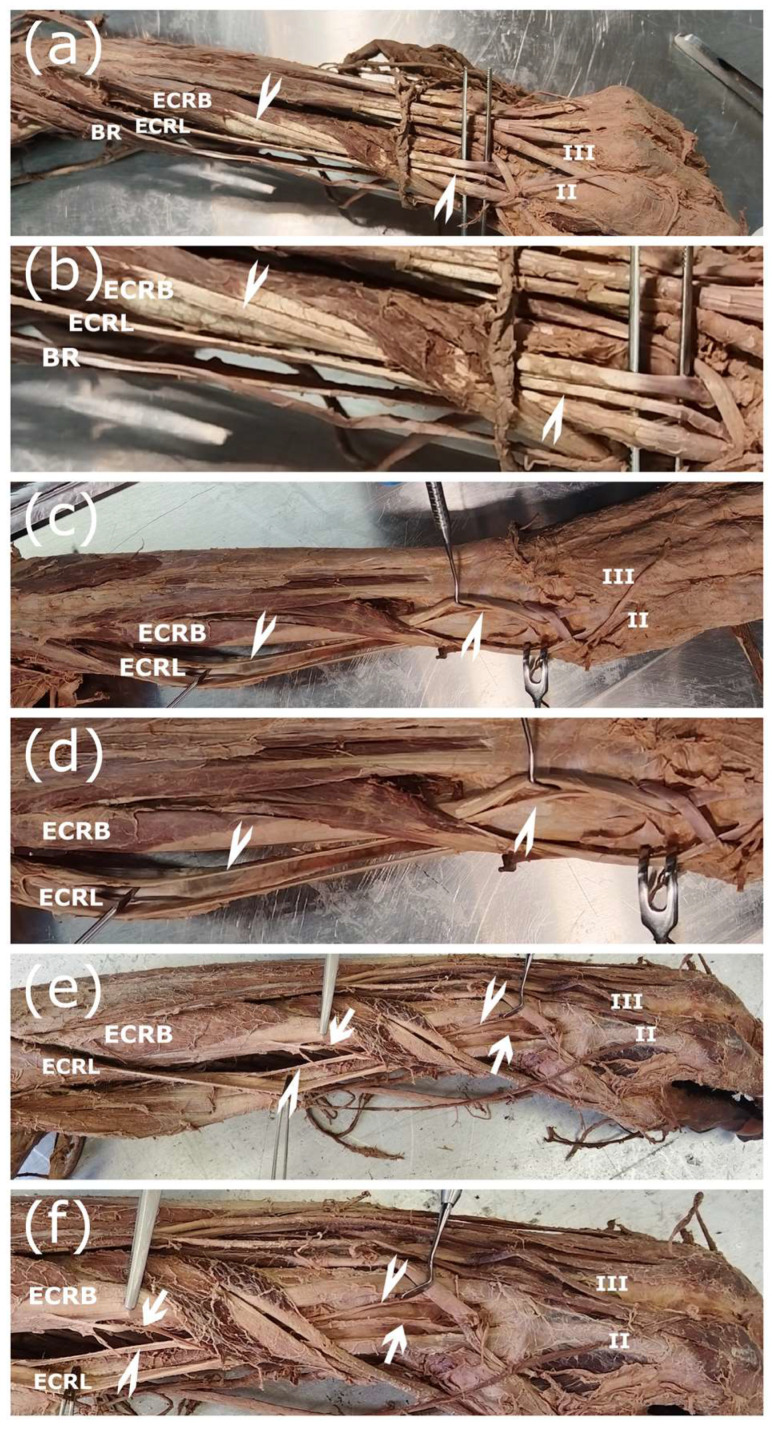
Interconnecting tendons between ECRL and ECRB. (**a**,**b**) The communicating tendon extends from the ECRB to the ECRL (arrowheads). Note that the tendon inserts medially to the main ECRL tendon (left arm); (**c**,**d**) The communicating tendon extends from the ECRL to the ECRB (arrowheads) (left arm). Note that the tendon inserts ventrally (underneath) to the main ECRB tendon; (**e**,**f**) Communicating tendons extend from the ECRL to the ECRB (arrowheads), and vice versa (arrows) (left arm). Note that the communicating tendons insert similarly to their solitary counterparts. BR—m. brachioradialis, ECRL—m. extensor carpi radialis longus, ECRB—m. extensor carpi radialis brevis, II—second metacarpal bone, III—third metacarpal bone.

Extra attention was directed toward the region between the ECRL and the ECRB, since certain interconnecting tendons can be extremely thin and are easily damaged during the dissection process. After dissecting the lateral compartment of the forearm, the number of supernumerary tendons was counted, and their attachment points were traced. Among the 58 limbs examined, supernumerary tendons were identified in 16 specimens (27.6%). Within the supernumerary group, four exhibited a connection emerging from the ECRL and ending at the ECRB (L-to-B), another four specimens displayed a connection originating from the ECRB and inserting into the ECRL (B-to-L), and the remaining eight specimens presented with both connections simultaneously (Both). For all L-to-B variations, the connection was attached specifically to the ventral side of the ERCB’s main tendon. Conversely, B-to-L connections terminated exclusively on the medial side of the ECRL’s main tendon. Examination of the origin of these interconnecting tendons revealed that the ECRL accessory tendon always originated from the ventral aspect of its body, whereas the ECRB originated from the midpoint of the muscle’s lateral aspect (Figure 3). These interconnecting tendons encompass a broad spectrum, ranging from fine tendinous slips to well-defined tendons. Sometimes part of the muscle belly separated and emerged as a supernumerary muscle belly. Regardless of the exact configuration, the described origin and insertion patterns were consistent.

### 3.3. Aberrant Fusion to Adjacent Muscle Groups

**Figure 4:**
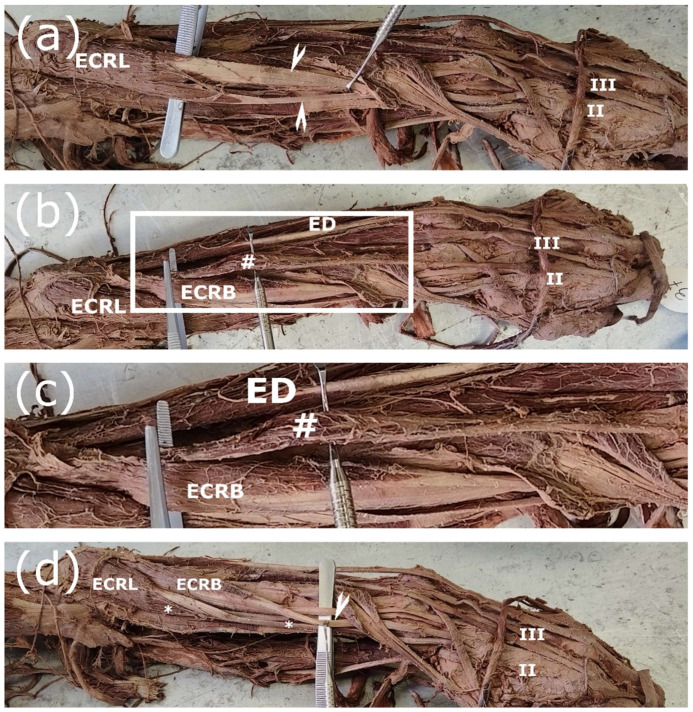
Aberrant fusion to m. extensor digitorum and bifurcation of both the ECRL and the ECRB (left arm). (**a**) A supernumerary tendon is observed arising from the ECRL muscle (arrowheads), resulting in a symmetrical split of the muscle body; (**b**,**c**) demonstrate that the extensor digitorum component for the index finger originates from the ECRB. White frame is enlarged in (**c**) for more detail. (**d**) The ECRB also bifurcates symmetrically, forming two separate muscle bodies and their corresponding tendons. ED—m. extensor digitorum, ECRL—m. extensor carpi radialis longus, ECRB—m. extensor carpi radialis brevis, II—second metacarpal bone, III—third metacarpal bone, #—the extensor digitorum portion for the index finger, *—show the position of the two parts of the ECRL.

As demonstrated in Figure 4b,c, we identified a rare variation: m. extensor indicis originating from the ECRB muscle belly. This specimen also presented a unique, extreme case of interconnecting tendons, where the muscle bodies of the ECRL and the ECRB bifurcated, and the supernumerary tendons were attached as previously described (Figure 4a,d). Furthermore, this specimen represents the sole instance in our study where a supernumerary B-to-L tendon originated from its dorsal aspect (Figure 4d).

### 3.4. Position of the Foetal Lateral Radial Extensor

**Figure 5:**
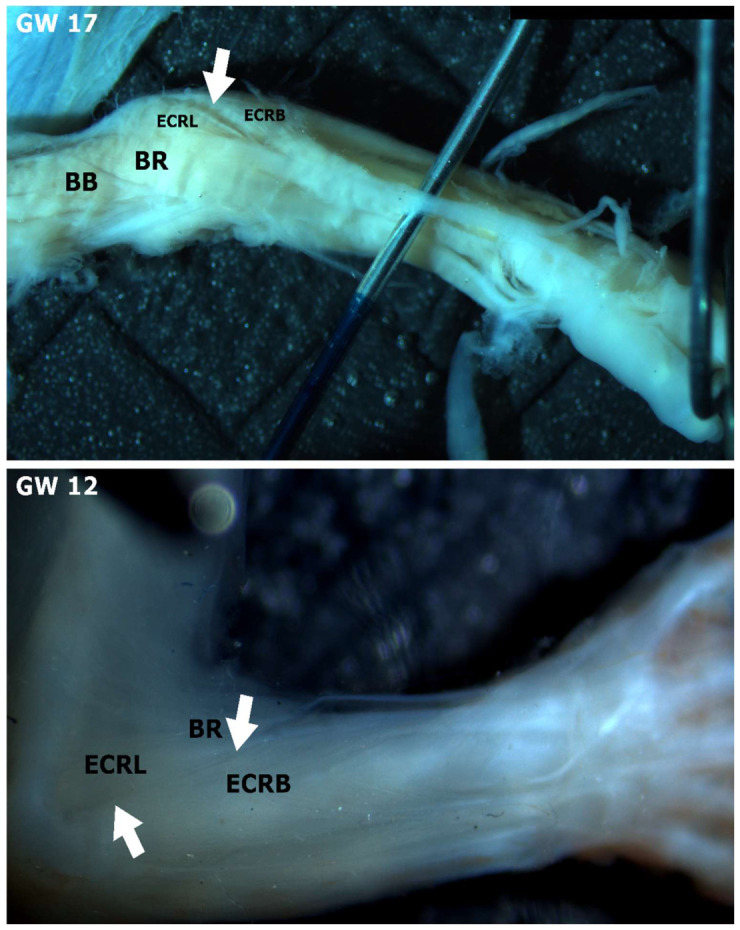
(GW 17) The radial carpal extensors are located at the lateral aspect of the forearm (left arm). (GW 12) (right arm). BB—m. biceps brachii, BR—m. brachioradialis, ECRL—m. extensor carpi radialis longus, ECRB—m. extensor carpi radialis brevis, arrows—indicate fission grove of lateral wrist extensors.

To investigate whether there are any differences in the macroscopic anatomy of the lateral wrist extensors between foetal and adult specimens, we performed dissections on two foetal forearms, one at GW 17 and one at GW 12. The forearm at GW 17 had well-established lateral wrist extensors. At GW 12, the lateral wrist extensors were also visible, but the segregation groove was significantly less pronounced (Figure 5). At GW 17, we observed that the separation between the ECRL and the ECRB was clearly pronounced in the proximal portion of the muscle (Figure 5). The lateral position of the two muscles, as well as their origin from the lateral epicondyle region, were evident in both dissections, similarly to the adult forearms.

### 3.5. Calculated vs. Observed Incidence of Single and Double Supernumerary Tendons

**Figure 6:**
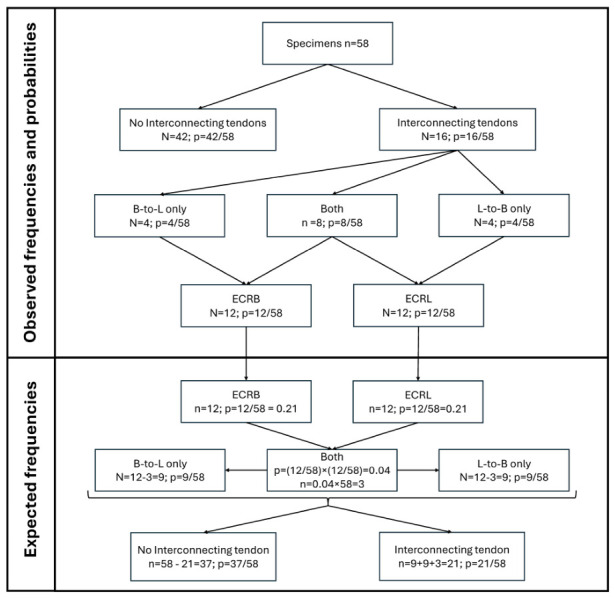
Steps for calculating observed versus expected probability of both interconnecting tendons occurring in the same upper limb. The observed frequencies of the four events—“No interconnecting tendon”, “interconnecting tendon longus-to-brevis only” (L-to-B only), “interconnecting tendon brevis-to-longus-only” (B-to-L only) and “interconnecting tendons on both muscles” (both), are compared to the distribution of a null hypothesis that the presence of an interconnecting tendon on either muscle is an independent event. This null distribution is calculated starting from the observation sample (n = 58) and the observed frequencies of interconnecting tendons on either muscle (12/58 for ‘L-to-B’ and 12/58 for ‘B-to-L’). Thus, the expected number of both interconnecting tendons occurring in the same arm is equal to 12/58 × 12/58 × 58 ≈ 2.3 ≈ 3. We chose to round the expected number up as a statistically more conservative approach. The expected frequencies of all other events are calculated from this number (see Section 2.3).

Next, we explored if statistical analysis of the distribution of these interconnecting tendons can give us some idea about their development. Regarding tendon formation, if we distance ourselves from all published data, there are few theoretically possible hypotheses: formation of a “dedicated” tendon for each muscle or fission of a common tendon precursor. Considering the observed distribution of supernumerary tendons, it is possible to relate the probabilities to the different modes of development. The observed frequencies and corresponding probabilities, associated with observing accessory interconnecting tendons from B to L, from L to B, both occurring at the same time (Both), and none, are shown in Figure 6. We suggest that if muscles and their tendons develop separately or by reduction of a pre-existing superfluous number of tendon “candidates”, each interconnecting tendon would be an independent event, while if they are formed by resorption of a preexisting common muscle/tendon primordium, the events would be mutually dependent.

In the case that the formation of accessory tendons of the radial carpal extensors is a random independent event, the expected distribution can be calculated from the observed frequency and probabilities following the rule for the co-occurrence of two independent events. To test this, we chose a simple χ^2^ goodness of fit test with as few assumptions as possible. Namely, we decided to compare the observed distributions of the four events—“no accessory tendons”, “conjoining tendon longus-to-brevis only” (L-to-B only), “conjoining tendon brevis-to-longus-only” (B-to-L only) and “conjoining tendons on both muscles” (both)—to the distributions of a null hypothesis that the presence of a conjoining tendon on either muscle is an independent event. Under this assumption of independence, the probability of two events occurring at the same time is:

P(A&“B′”) = P(“A”) × P(“B”|“A”) = P(“B”) × P(“A”|“B”) = P(“A′”) × P(“B′”)

Using the observed frequencies and this rule, we could calculate the expected distribution of the null hypothesis of independent events (Figure 6 and Table 1). We calculated the null distribution starting from our observation sample (n = 58) and the observed frequencies of conjoining tendon on either muscle (12/58 for ‘L-to-B’ and 12/58 for ‘B-to-L’). Starting from this, the expected number of both conjoint tendons occurring in the same arm is equal to 12/58 × 12/58 × 58 ≈ 2.3 ≈ 3. We chose to round the expected number up (instead of down as mathematically appropriate) as the more conservative approach and for additional protection against type I error. Correspondingly, the expected number of only one conjoint tendon occurring on the ECRL (‘L-to-B only’) is 9 (‘L-to-B’—Both = 12 − 3), and the opposite ‘B-to-L only’ is also 9 (‘L-to-B’—Both = 12 − 3). Finally, the expected number of no conjoint tendons remains 58 − (9 + 9 + 3) = 37.

Since the expected frequency of the joint event is very low, we used the chi-square test with a simulated *p*-value based on 2000 replicates. The goodness-of-fit test of the observed versus the expected distribution showed a significant difference (χ^2^(3) = 14.565; *p* = 0.0009995), rejecting the null hypothesis that the formation of ECRL and ECRB accessory tendons are completely random, mutually independent events. The high incidence of both interconnecting tendons present is more consistent with the two events being part of a common process, thus increasing the likelihood of the hypothesis that the two muscle tendons have a common origin and are generated by fission, which is consistent with the literature [3,11,16].

### 3.6. Hypothetical Mismatch Between the Division Planes of the Muscle and Tendon Primordium Could Explain the Observed Morphology of Interconnecting Tendons

A good theoretical explanation on the origin of interconnecting tendons should take into account several points: 1. The common origin of the lateral compartment muscles and their relatively late separation; 2. The distribution of single versus double interconnecting tendons suggests that they are not independent events, as does the literature; 3. L-to-B always inserts under ECRB, while B-to-L always inserts ulnar to ECRL; 4. L-to-B emerges from the ventral portion of the ECRL muscle body, while B-to-L emerges almost always from the midportion of the ECRB muscle body; 5. According to the literature, proximal tendon splitting is muscle-dependent.

**Figure 7:**
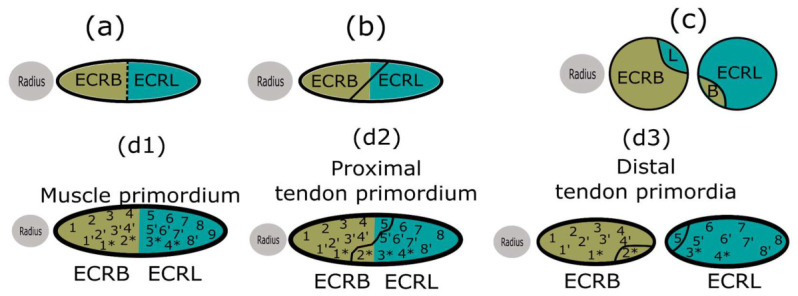
(**a**) A graphical representation of the common lateral wrist extensor primordium with the putative division line. (**b**,**c**) When the division line is oblique, parts of the muscle can be exchanged. (**d1**) The putative positions of muscle cell bundles in the common primordium of the radial wrist extensors, named with arbitrary placeholder numbers. (**d2**) The corresponding tendon bundles of the common tendinous precursor that would develop into the topographic muscle–tendon junctions are presented, along with the same placeholder numbers. Note how the putative border between the two tendons here is a straight line. (**d3**) One can see that the ventral part of the ECRB tendon (marked with 2*) ends up attached to the body of the ECRL, and the medial part of the ECRL tendon (marked with 5) ends up attached to the body of the ECRB. ECRL—m. extensor carpi radialis longus, ECRB—m. extensor carpi radialis brevis, L—remnant from longus, B—remnant from brevis. *—mark putative ventral bundles in the tendon.

Here we propose a plausible hypothesis that considers all the above and is summarized in Figure 7. When the common muscle primordium splits, the fission plane might not be aligned, but is oblique in relation to the already pre-existing distal tendon fission plane, so that the “part of ECRL” body will stay with the brevis and vice versa (Figure 7a–c). Nevertheless, the positions of the interconnecting tendons described in points 3 and 4 remain unaddressed. We propose that the muscle and tendon primordia could connect in a particular topographic manner, and not randomly. Thus, when separation begins after the oblique division of the muscle body, it should take along with it several of the tendon slips that were destined to become a part of the main tendon of the opposite muscle (Figure 7(d1–d3)). Of course, only a single communicating tendon might be present. Then this “oblique” division line could occur only in the upper or lower portion of the common muscle primordium. However, since two interconnecting tendons are more common, it seems that a complete oblique division occurs more often. It is logically sound that the dimensions of the interconnecting tendons should depend on the degree of the fission line mismatch.

## 4. Discussion

Beyond the clinical setting, anatomical variations encountered in the dissection hall offer a compelling way to introduce medical students to the concept that “normal anatomy” comprises a spectrum of possible arrangements, particularly when complex and unique cases arise [17,18]. This is especially significant given that approximately 10% of medical malpractice cases are attributed to an underlying anatomical variation [19,20]. Recent reports suggest an even higher incidence, indicating that anatomical variations are a factor in up to 25% of all medical malpractice claims in the US [21]. The accurate systematic documentation of anatomical variations is of high clinical relevance. In the present case, variations in ECRL and ECRB, especially in their supernumerary tendons, have been extensively studied due to their importance in surgical procedures, such as tendon transfers used to restore partial functionality in paralytic patients [14,22]. Introducing the notion of anatomical variability early in medical education ensures it becomes an integral component of a medical student’s clinical reasoning. While the clinical and educational significance of these variations is clear, a proper mechanistic explanation for the formation of interconnecting tendons remains elusive. Recent studies have investigated the musculotendinous junction, employed lineage tracing to determine cellular origins, or examined the junctions between bone and muscle and their patterning across the three primary body axes [23,24,25]. Several studies characterize the descriptive anatomy of muscle development, the timing of muscle formation or the stages of muscle maturation [11,26,27,28]. To our knowledge, no studies have yet sought to explain the mechanisms by which muscle variations emerge. This likely stems from the fact that their variability and low probability of observation make experimental design exceptionally complicated and difficult to approach. This challenge is reflected in the gaps in our understanding of the development and morphogenesis of specific muscles. In the present study, we focused on the variability of the ECRL and ECRB. Current data show that the ECRL and ECRB develop as one muscle mass, separating from each other around GW 9 [26]. Muscle splitting starts in the proximal part and proceeds distally [3]. During the splitting process, connective tissue gradually accumulates at the initial cleavage site and slowly replaces the muscle cells in the region, which undergo cell death [3]. Tendon precursors arise from the lateral plate mesoderm, while muscle precursors arise from the dermatomyotomes [2,3,4,29]. In their distal portion, developing tendons undergo splitting to form separate tendons, while the proximal part relies on attachment to the newly formed muscles and subsequent movement for both survival and proper fission [3,12,13,27]. In the adult forearm, the ECRL and ECRB have separate origins, while the ECRB and ED (m. extensor digitorum) start from a common osteotendinous junction [30,31]. This is consistent with the common extensor muscle mass during early development, potentially explaining the fusion between ECRB and EDC (m. extensor digitorum communis) observed both in our study and in previous literature [32]. Our findings reveal two main variation types: failed muscle division and remnants of an improper division.

### 4.1. Interconnecting Tendons

The supernumerary tendons of the lateral wrist extensors have been well studied due to their clinical importance. They cross over and connect to the adjacent radial wrist extensor muscle [14]. Our observations show that L-to-B interconnecting tendons attach to the OM III, always ventrally to the ECRB main tendon and B-to-L terminate at OM II, located on the ulnar side of the ECRL main tendon. Our results seem to be in accordance with Albright and Linburg (1978) [14]. Such interconnections range between 35% and 7.8% in the literature [14,33]. We found no previous reports providing a detailed description of the muscle origins associated with the interconnecting tendons. Notably, we found one atypical case, in which the delamination process extended beyond the middle portion of ECRB to include the entire proximal half, forming an unusually large B-to-L interconnecting tendon. Furthermore, the interconnecting tendons could be accompanied by incomplete segregation of the muscle bodies, forming a partial accessory belly. We suggest caution in classifying these variations as m. extensor carpi radialis accessorius (ECRA) or m. extensor carpi radialis intermedius (ECRI) since, according to their classical descriptions, they possess distinct attachment points and positions relative to the main muscle body [34,35,36].

### 4.2. Distribution of Interconnecting Tendons

In this study, we employed a statistical analysis of the interconnecting tendon distribution to determine whether the systematic evaluation of anatomical variations can be a reliable tool for assessing developmental processes. We propose that if the formation of the ECRL and ECRB tendons occurs independently, the presence of both interconnecting tendons in a single limb would be expected to be rare, relative to the appearance of a single interconnection. However, we observed the opposite phenomenon and the distribution analysis we performed suggests that the formation of interconnecting tendons involves interdependent events. Our observations and conclusions are supported by the literature, since both the radial wrist extensors and their tendons diverge from a common primordium [11,26].

### 4.3. Plausible Hypothesis for the Emergence of Interconnecting Tendons

At first glance, the occurrence of supernumerary muscle or tendon slips seems easy and straightforward to explain: aberrant patterning leads to additional schism spots in the primordial muscle bellies. However, accessory tendons display a highly consistent recurring insertion pattern, which has not been addressed from a developmental perspective. Here, we propose that an oblique muscle division and a mismatch in the putative division plane of the tendon could account for nearly all characteristics of the interconnecting tendons. As previously noted, we lacked a definitive explanation for the consistency in the anatomical origins of these variable tendons and speculated that certain migratory processes might be responsible. An extensive literature review revealed two reports that support this hypothesis. One researcher described the migration of the ECRL and ECRB, after their initial formation in the posterior aspect of the forearm, to the lateral aspect [37]. One can also observe some positional differences in the images submitted by Wilde et al. (2020) [28]. The authors did not comment on these themselves, so this is rather an interpretation of the submitted data [28]. According to Gräfenberg (1905) [37], this migration occurs around GW 9. This aligns with our own observations, as we found the musculature already in a lateral position by GW 12.

### 4.4. Failed Division of the Muscle Bodies

It is possible for ECRB to fuse with ED, either completely or partially, or by arising from its proximal fascia or tendon, probably due to the common extensor primordium during development [1,32]. After the initial cleavage of the extensor muscle group from the common extensor primordium, ECRL and ECRB continue their development as a single muscle much longer than the others and separate at early GW 9 [26,28]. The muscle bodies of ECRL and ECRB body that gives rise to one tendon. More distally, this tendon will split into two or three parts, inserting as normal on the OM II and OM III [1,38]. In some cases, the common tendon will split asymmetrically, with a significantly smaller tendon going towards the OM III, replacing the missing ECRB tendon [1]. Most authors present the case of a common extensor carpi radialis body as ECRL muscle agenesis [1,38]. We observed that the common muscle body originated from both the lateral epicondyle and the lateral epicondyle ridge, the expected origins of ECRB and ECRL, respectively. We suggest that this is probably due to failed muscle divisions accompanied by the muscle-independent development of the proximal parts of limb tendons [3,11,12,13]. Referring to the case as a failed division, rather than agenesis, would be more accurate in our opinion. It is worth mentioning that the previous authors did not discuss the origin of the common muscle belly. An example of a molecular basis for failure of muscle bodies to divide was proposed by Swinehart et al. (2013) [32]. They demonstrate that mutant mice lacking the *Hoxa11/d11* genes show severe muscle patterning defects in the zeugopod (analogous to the forearm of adult humans). One of the many observed defects is the failure of ECRL, ECRB and ED to divide, so at E14.5, they remain a common muscle mass [32].

### 4.5. Clinical Relevance

Tendon transfer from the ECRL or ECRB can be performed for partial restoration of wrist movement in paralytic patients [14,22,39]. Supernumerary tendons of the ECRL and ECRB have been used to this end, without completely sacrificing the function of the relevant muscle. We again want to stress, as mentioned above, that the ECRA and ECRI are different from the interconnecting tendons we report here [34,35,36]. The ECRL can be used also for tendon transfer to the finger flexors or m. extensor carpi ulnaris (ECU) to restore function [39,40,41]. If an additional, well-formed belly is present, as seen in Figure 4, it is always connected to the L-to-B interconnecting tendon, according to our observations. In this case, it is possible that the L-to-B transferring might be more beneficial for flexor transfer, since it is located on the ventral side, while the main tendon of the ECRL is more appropriate for ECU transfer. The main reasoning is that the angles and the location of the muscle bellies are at more mechanically sound positions during contraction and no shear forces would apply.

### 4.6. Future Work and Study Limitations

The study is limited by the lack of experimental confirmation to our putative model. Although the foetal upper limb sample is small, it should allow us to see major positional changes in the ECRL and ECRB muscles if present. The lack of observable positional differences between the adult and foetal arms is consistent with other literature as well. It is worth noting that smaller deviations between adult and foetal arms require bigger samples and more precise techniques. The other major limitation is the lack of earlier time periods due to clinical constraints—abortions before GW 12 are usually performed by curettage, which does not allow for morphology preservation. Fifty-eight limbs is also a rather small sample and should be expanded in future research on the topic. To test our putative model, a histological study of early foetal arms should be considered, like sections conducted by other groups [11]. The chance of both interconnecting tendons being present at the same time is rather low (8/58 or 13.7%). Although visualisation of the tendon fission plane is achievable with both immunohistochemistry and histochemistry, the low probability of variation would require large amounts of foetal specimens. Obtaining them in sufficient quantities is challenging. Another possible and less invasive way to study human foetal development is provided by micro-CT scans [42]. Newer models can achieve a resolution of up to 1 µm, which should be enough to visualise the fission plane angle, especially when paired with connective tissue contrast [43,44]. Additionally, a meta-analysis of existing literature might provide enough data for the formulation and testing of more detailed hypotheses.

## 5. Conclusions

Careful and systematic studies of anatomical variation in adult humans might be beneficial in building hypotheses on the development of the human body. Of course, the hypothesis should be further evaluated.

## Figures and Tables

**Table 1:** Describing the observed vs. predicted values of interconnecting tendon variations.

Variant	Observed		Expected	
	Frequency	Probability	Frequency	Probability
No accessory tendons	42	42/58	37	37/58
Brevis-to-Longus only (B-to-L only)	4	4/58	9	9/58
Longus-to-Brevis only (L-to-B only)	4	4/58	9	9/58
Both	8	8/58	3	3/58
Sum	58	1	1	1

## Data Availability

The data presented in this study is available on request from the corresponding author. Data is not publicly released to protect the privacy and anonymity of the donors.

## Abbreviations

The following abbreviations are used in this manuscript:

GW	gestational week
OM II/OM III	os metacarpi II/III
ECRL	extensor carpi radialis longus
ECRB	extensor carpi radialis brevis
BR	brachioradialis
ED	extensor digitorum
BB	biceps brachii
L-to-B	longus to brevis
B-to-L	brevis to longus
